# The genetic polymorphisms at the promoter region of *HLA-DQB1 *gene, creating responsive elements for NF1/CTF and converting the TFII-D binding site to GR-alpha

**DOI:** 10.22099/mbrc.2023.46890.1813

**Published:** 2023

**Authors:** Khyber Saify

**Affiliations:** Department of Biology, College of Education Sciences, Kunduz University, Kunduz, Afghanistan

**Keywords:** GR-alpha, HLA-DQB1, NF1/CTF, Polymorphism

## Abstract

Human leukocyte antigen-DQB1 (HLA-DQB1, OMIM: 604305) is the human major histocompatibility complex (MHC) system. HLA genes are classified into three classes (I, II, and III). The HLA-DQB1 belongs to class II, is mainly involved in the actions of the human immune system and plays a fundamental role in donor-recipient matching in transplantation and can be associated with most autoimmune diseases. In this study, the potential influence(s) of the G-71C (rs71542466) and T-80C (rs9274529) genetic polymorphisms were investigated. These polymorphisms, located in the *HLA-DQB1* promoter region, have a significant frequency in the world population. The online software ALGGEN-PROMO.v8.3 was used in this work. The results indicate that the C allele at the -71 position actually creates a new potential binding site for NF1/CTF and the C allele at the -80 position changes the TFII-D binding site into a GR-alpha response element. The NF1/CTF plays the role of activator and the GR-alpha is the inhibitor; thus, according to the roles of these transcription factors, it is suggested that the above-mentioned polymorphisms alter the expression levels of *HLA-DQB1*. Therefore, this genetic variation is associated with autoimmune diseases; however, this cannot be generalized because this is the first report and more studies are needed in the future.

## INTRODUCTION

In fact, the human major histocompatibility complex (MHC) is another name for the human leukocyte antigen (HLA) system [1]. The HLA complex of genes, actually encodes proteins that are centrally involved in the actions of the human immune system. HLA genes are divided into three categories (I, II and III). HLA-DQB1 belongs to the second category [1, 2]. The class II molecule is a heterodimer that includes an alpha chain (DQA) and a beta chain (DQB), both of which are attached to the membrane. It plays a necessary role in the immune system by presenting peptides derived from extracellular proteins produced by foreign invaders such as viruses and bacteria and presentation to CD4+ and helper T cells. 

The *HLA-DQB1* located on the short arm of human chromosome 6 (6p21.3). Previous studies have shown that this human chromosomal region is associated with several multifactorial diseases such as schizophrenia, Alzheimer's disease, pre-eclampsia and gastric cancer [3-6], indicating that polymorphisms in the *HLA-DQB1* might be involved in some multifactorial traits. Previous genetic association studies have shown that *HLA-DQB1* and its polymorphisms and allelic variation play an important role in donor-recipient matching in transplantation [1, 2, 7] and may be associated with autoimmune diseases such as type I diabetes, multiple sclerosis (MS), celiac disease [8], viral infections [9], hepatitis C [10], hepatitis B, cirrhosis [11], ovarian cancer, breast cancer cases with ataxia [12], and childhood steroid-sensitive nephrotic syndrome [13]. In this study, we analyzed the G-71C (rs71542466) and T-80C (rs9274529) genetic polymorphisms in the promoter region *of HLA-DQB1* and their effects on transcription factor binding sites.

## MATERIALS AND METHODS

This investigation was conducted using the method of our previous study [14]. First, the promoter sequence of the HLA-DQB1 gene was obtained from NCBI. Second, the polymorphic sites (rs71542466) and (rs9274529) were identified on the sequence. The distribution and allele frequencies of these polymorphisms in different populations were investigated using the NCBI online database and are shown in Table 1. 

To identify the transcription factor binding site, ALGGEN-PROMO.v8.3 online software was used (https://alggen.lsi.upc.es/cgi-bin/promo_v3/promo/promoinit.cgi?dirDB=TF_8.3). The wild-type and variant alleles were analyzed separately to identify the location of the transcription factor; in the whole process of analysis, the maximum matrix dissimilarity rate was assumed to be 3%. It should be noted that in rs9274529, T changes to C or T changes to A, since the frequency of A alleles in the worldwide populations is equal to zero, therefore it was not included in the analyses performed in the current research.

**Table 1:** Allelic distribution of genetic polymorphisms (rs71542466) and (rs9274529) promoter region of *HLA-DQB1* in the worldwide populations

**Population**	**Allelic frequency (rs71542466)**				**Allelic frequency (rs9274529)**		
	**Simple Size**	**G(%)**	**C(%)**		**Simple Size**	**T (%)**	**C (%)**
European	12080	84.0	16.0		14152	40.5	59.5
African	2816	79.2	20.8		2898	49.9	50.1
African Others	108	73.1	26.9		114	54.4	45.6
African American	2708	79.5	20.5		2784	49.8	50.2
Asian	108	78.7	21.3		112	38.5	61.5
East Asian	84	82.0	18.0		86	37.0	63.0
Other Asian	24	67.0	33.0		26	42.0	58.0
Latin American 1	146	80.1	19.9		146	41.8	58.2
Latin American 2	610	87.5	12.5		610	27.9	72.1
South Asian	94	72.0	28.0		98	50.0	50.0
Other	478	81.0	19.0		504	40.5	59.5
**Total**	**19256**	**78.6**	**21.4**		**21530**	**42.9**	**57.1**

It should be noted that in the rs9274529 T changes to C or T changes to A(T>C or T>A), since the frequency of A alleles in the world population is equal to zero, it was not included in the analyses (https://www.ncbi.nlm.nih.gov/snp/rs71542466 and https://www.ncbi.nlm.nih.gov/snp/rs9274529).

## RESULTS AND DISCUSSION

**Figure 1:**
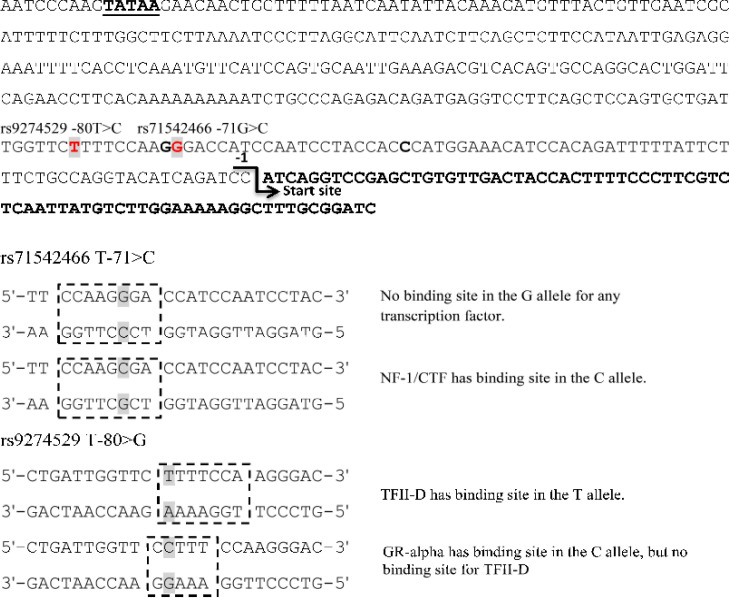
Genetic polymorphisms in the promoter region of the human leukocyte antigen-DQB1 (*HLA-DQB1*) gene and its influence on transcription factor recognition sites

The allelic frequencies of rs71542466 and rs9274529 polymorphisms among the worldwide populations are shown in (Table 1). Both polymorphisms are the more common allele in the worldwide populations. The results show that the -71G>C substitution in the promoter of *HLA-DQB1* creates a new potential NF1/CTF responsive element (CCAAGGGA changed to CCAAGCGA) (Fig. 1). Also, the C allele at position -80T>C causes the TFII-D binding site (TTTTCCA) to change to GR-alpha (CTTTCCA), which acts as a responsive element (Fig. 1).

This study shows that the -71G>C substitution in the promoter of *HLA-DQB1* forms a new potential NF1/CTF responsive element (Fig. 1). Nuclear factor I (NFI) is a subcategory of site-specific DNA-binding proteins involved in viral DNA replication and regulation of gene expression. [15]. The NF1 transcription factor is known to play an important activating role [16]. Our results show that NF1/CTF binds to the *HLA-DQB1* promoter only when the C allele is present at the -71 position, but the G allele does not have a binding site for this transcription factor. Considering the "activator" role of NF1/CTF transcription factor, the C allele is expected to increase the transcriptional activity of *HLA-DQB1*.

The GR-alpha is a highly conserved multifunctional transcription factor and some studies explain that when it is bound to the promoter sequence of MHC class II genes, it results in the down-regulation of MHC class II expression [17-19]. It should be that HLA-DRB1 is the member of MHC class II [1, 2]. It is suggested that the conversion in the elements of TFII-D into GR-alpha could alter the expression of *HLA-DQB1* as TFII-D is one of the overall transcription factor playing a core role in the first stage of transcription [20]. The T allele at the -80 position is the most notable allele in the worldwide populations. The results of the present study show that the TFII-D is able to bind to the promoter region in the presence of the T allele at -80 positions. Thus, in the presence of C allele at -80 position, TFII-D substitutes its respective position for GR-alpha. According to the role of GR-alpha, which is an inhibitor; the C allele can down regulate the transcriptional activity of the gene *HLA-DQB1*.

This is the first research showing that the G-71C and T-80C polymorphisms create new putative NF1/CTF and GR-alpha binding sites, respectively. Some studies conclude that genetic polymorphisms are not similar among ethnic groups. The polymorphisms investigated in the current study follow the same situation. The literature also suggests that the polymorphisms of *HLA-DQB1* are significantly associated with the autoimmune diseases such as type I diabetes, multiple sclerosis, celiac disease, viral infection, hepatitis C, hepatitis B, liver cirrhosis, ovarian cancer, breast cancer with ataxia, and steroid-sensitive nephrotic syndrome in children. It also plays a key role in donor-recipient matching in transplantation. [1, 2, 7-13]. In addition, previous studies conclude that single nucleotide polymorphisms in the promoter region can cause a change in the expression of the gene [21]. The polymorphisms in this study suggest that they modify the risk of autoimmune diseases; however, this cannot be generalized because this is the first report and more studies are needed in the future.

### Acknowledgements:

The author is indebted to assistant professor Mr Mohamad Akmal Saifi M.ED (TESL), for critical reading of the manuscript and also the cooperation of the Kunduz University administration is much appreciated.

### Conflict of Interest:

No competing interests are declared by author.
